# Editorial: MINOCA: Pathogenesis, Diagnosis, Clinical Management and Evolution Towards Precision Medicine

**DOI:** 10.3389/fcvm.2022.928515

**Published:** 2022-06-14

**Authors:** Domenico D'Amario, Rocco A. Montone, Josip A. Borovac

**Affiliations:** ^1^Department of Cardiovascular and Thoracic Sciences, Istituto Di Ricovero e Cura a Carattere Scientifico Fondazione Policlinico A. Gemelli, Universita Cattolica Sacro Cuore, Rome, Italy; ^2^Clinic for Heart and Vascular Diseases, University Hospital of Split, Split, Croatia; ^3^Department of Pathophysiology, University of Split School of Medicine, Split, Croatia

**Keywords:** MINOCA, myocardial infarction without obstructive coronary disease, percutaneous coronary intervention, risk factors, imaging, antiplatelet therapy (AT), cardiac magnetic resonance (CMR) imaging, invasive functional testing

Myocardial infarction with non-obstructive coronary disease (MINOCA) represents a heterogeneous clinical conundrum, accounting for approximately 6% of all presentations of acute myocardial infarction (AMI) (1). The diagnosis of MINOCA is often challenging in clinical practice; however, traditional criteria for AMI, as defined by the fourth universal definition of myocardial infarction (UDMI), outline that it is accompanied by no stenosis ≥50% in a major coronary artery on coronary angiography (2, 3). The pathophysiological mechanisms in MINOCA are still poorly elucidated and may originate from the processes occurring in the epicardial coronary vessel, in the form of rupture or a fissure of small atherosclerotic plaques, spontaneous coronary artery dissection (SCAD), coronary vasospasm, or *in situ* thrombosis (4). Furthermore, coronary microvascular disease (CMD) and increased oxygen demand-to-supply ratio, i.e., type 2 AMI without significant CAD on angiography might be the underlying mechanisms behind MINOCA (5). Compared to patients with obstructive coronary artery disease (CAD) AMI, patients with MINOCA are slightly younger, more commonly women, and generally have fewer traditional cardiovascular disease (CVD) risk factors or positive history of CVD (4). From a prognostic standpoint, it was initially thought that MINOCA is a benign condition associated with a favorable prognosis, although recent data clearly show that MINOCA is associated with a significant risk of long-term mortality, reinfarction, and major adverse cardiovascular events (MACE) (6–8).

To better understand the mechanisms behind MINOCA, improve clinical outcomes, and reduce healthcare-related costs, it is necessary to foster research efforts with the potential to unravel the specific causes of MINOCA, meaning therapeutic management can be specifically tailored toward the underlying culprit mechanism (9). For these reasons, this Research Topic on MINOCA aggregated relevant original and review articles that explore this pathophysiological entity from the perspective of diagnosis, prognosis, and treatment.

Concerning diagnosis, Mangiacapra et al. cover the landscape of unmet clinical needs in MINOCA, examining the invasive functional coronary assessment of patients presenting with MINOCA. In these patients, vasomotion abnormalities and CMD might play an important causal role, requiring a comprehensive functional assessment of both epicardial and microvascular coronary circulation. The authors give a relevant overview of the definition and pathogenesis of MINOCA and continue examining invasive cath-lab methods and provocative tests that can yield functional and prognostic information in patients with MINOCA. Finally, they briefly describe an etiology-tailored therapeutic strategy based on a comprehensive invasive evaluation of the pathophysiological mechanisms of ischemia in MINOCA patients.

As MINOCA often presents a formidable diagnostic challenge to clinicians, clinical practices require novel imaging methods that can distinguish MINOCA from other non-ischemic etiologies of myocardial injury such as acute myocarditis, Takotsubo syndrome, and other conditions (10). In their article, Liang et al. discuss the value and the role of cardiac magnetic resonance (CMR) imaging in the diagnosis of MINOCA. The authors examine the use of CMR in MINOCA from the perspective of current international guidelines, describe relevant morphological and functional disease patterns that might aid in MINOCA diagnosis, discuss potential MINOCA mimics that are frequently encountered in clinical practice, and finally discuss limitations and future outlooks of the CMR as an important diagnostic modality for this disease entity.

The bidirectional relationship between sleep disorders and poor clinical outcomes has been well-established among patients with CAD (11, 12). However, sleep disorders have not been previously examined in the population of patients with MINOCA and were not analyzed with respect to new MACE occurrence after the index event. In their work, encompassing 607 Chinese outpatients with MINOCA that were followed for a mean follow-up of nearly 4 years, Zhu et al. demonstrated that poor sleep quality and short sleep duration were independent predictors of all-cause mortality and MACE in the MINOCA population, thus stressing that mitigation of poor sleep patterns is likely an important secondary cardiovascular prevention measure in this population.

Patients with MINOCA exhibit an annual rate of total long-term mortality of 2.2% compared to 5.0% in patients with obstructive AMI, however, such a rate is not negligible and calls for clinical attention and devisement of adequate treatment plans (6). Furthermore, in some populations, diagnosis of MINOCA might have similar outcomes to obstructive AMI with respect to all-cause death, cardiac death, non-cardiac death, and reinfarction (13). Given its relevant impact on prognosis, Yin et al. evaluated the prognostic performance of the GRACE score in the setting of MINOCA due to the well-established value of such a risk stratification system among patients with non-ST-elevation myocardial infarction (NSTEMI). Their study enrolled 340 consecutive patients with NSTEMI and angiographic findings of non-obstructed coronary arteries. The authors show that the cardiac death at 1-year follow-up was significantly higher among patients that were classified as high-risk according to GRACE score, compared to the low-to-intermediate risk group. The incidence of MACE was significantly higher in patients with high GRACE scores compared to those with low GRACE risk scores. Authors, therefore, concluded that the calculation of the GRACE risk score might provide useful prognostic information with respect to clinical outcomes in MINOCA patients with NSTEMI, during the first year following hospital discharge.

The pharmacological treatment of MINOCA remains an open question, with some cohort-level data supporting the use of ACE inhibitors/ARBs, statins, and cardiac rehabilitation, after defining the underlying mechanism of clinical presentation (14). Due to the controversial role of antiplatelet agents in MINOCA, Ortega-Paz et al. focused on the role of antiplatelet therapy for this condition, a pharmacotherapeutic area that has not been clearly defined and is a subject of expert debate to date. In their work, the authors emphasize the importance of determining the etiology of MINOCA and making a clear distinction between atherosclerotic and non-atherosclerotic mechanisms of MINOCA with the possibility of antiplatelet therapy use in cases of the former scenario.

Finally, Merlo et al. present an overview of atypical risk factors and associated comorbidities that are more frequently encountered than traditional cardiovascular risk factors in patients with MINOCA compared to patients with classic obstructive myocardial infarction.

This Research Topic aimed to cover fundamental gaps in our knowledge of this relevant and heterogeneous clinical entity. Different definitions, lack of appropriate comparative populations, and different clinical and therapeutic approaches make it a comprehensive and agreed clinical algorithm, but it is still lacking. Major efforts should be undertaken, aiming to determine the specific cause for each patient in each setting, to personalize a targeted therapy. For educational purposes, it is fundamental that healthcare professionals are daily exposed to the complexity of the diagnosis and management of patients with MINOCA so that patients are appropriately identified and treated: “You may say I'm a dreamer, but I'm not the only one. I hope someday you'll join us”! (a saying by John Lennon).

## Author Contributions

DD'A, RM, and JB wrote the original draft of the Editorial and made critical revisions to the draft. All authors agree with the final version of the manuscript to be submitted.

## Conflict of Interest

The authors declare that the research was conducted in the absence of any commercial or financial relationships that could be construed as a potential conflict of interest.

## Publisher's Note

All claims expressed in this article are solely those of the authors and do not necessarily represent those of their affiliated organizations, or those of the publisher, the editors and the reviewers. Any product that may be evaluated in this article, or claim that may be made by its manufacturer, is not guaranteed or endorsed by the publisher.
